# *In Vitro* Evaluation of Antioxidant, Anticancer, and Anti-Inflammatory Activities of Ethanolic Leaf Extract of *Adenium obesum*

**DOI:** 10.3389/fphar.2022.847534

**Published:** 2022-07-19

**Authors:** Ahmad Alshehri, Afza Ahmad, Rohit Kumar Tiwari, Irfan Ahmad, Ali G. Alkhathami, Mohammad Y. Alshahrani, Mohammed A. Asiri, Tahani M. Almeleebia, Mohd Saeed, Dharmendra Kumar Yadav, Irfan Ahmad Ansari

**Affiliations:** ^1^ College of Applied Medical Sciences, Najran University, Najran, Saudi Arabia; ^2^ Department of Biosciences, Integral University, Luknow, India; ^3^ Department of Clinical Laboratory Sciences, College of Applied Medical Sciences, King Khalid University, Abha, Saudi Arabia; ^4^ Department of Clinical Pharmacy, College of Pharmacy, King Khalid University, Abha, Saudi Arabia; ^5^ Department of Biology, College of Sciences, University of Hail, Hail, Saudi Arabia; ^6^ Gachon Institute of Pharmaceutical Sciences and Department of Pharmacy, College of Pharmacy, Gachon University of Medicine and Science, Incheon, South Korea

**Keywords:** cytokines, anticancer, anti-inflammatory, antioxidant, TNF-α

## Abstract

*Adenium obesum* commonly known as “desert rose” belongs to the family Apopcynaceae and has previously been reported for its anti-influenza, antimicrobial, and cytotoxic efficacies and well-known for their ethno-medicinal applications. In the present study, ethanolic extracts of *A. obesum* (AOE) were analyzed by gas chromatography-mass spectrometry (GC–MS) to identify the important phytochemical compounds. The GC–MS analysis of AOE detected the presence of 26 phytochemical compounds. This plant is traditionally used for the treatment of various diseases. In this report, the antioxidant, anti-inflammatory, and anticancer activities of ethanolic leaf extract from *A. obesum* (AOE) were studied. The antioxidant potential of ethanolic extract of AOE was examined by different antioxidant assays, such as antioxidant capacity by the DPPH, ABTS, superoxide, hydroxyl radical scavenging, and lipid peroxidation inhibition assays. The antioxidant activities of various reaction mixtures of AOE were compared with a reference or standard antioxidant (ascorbic acid). In addition, we also evaluated the anticancer activity of AOE, and it was observed that AOE was found to be cytotoxic against A549 lung cancer cells. It was found that AOE inhibited the viability of A549 lung cancer cells by inducing nuclear condensation and fragmentation. Furthermore, ethanolic AOE demonstrated the anti-inflammatory potential of AOE in murine alveolar macrophages (J774A.1) as an *in vitro* model system. AOE showed its potential in reducing the levels of inflammatory mediators including the proinflammatory cytokines and TNF-α. The results obtained in the present investigation established the antioxidant, anticancer, and anti-inflammatory potency of AOE, which may account for subsequent studies in the formulation of herbal-based medicine.

## 1 Introduction

Since ancient times, many natural compounds isolated from different medicinal plants have been extensively used for the treatment of numerous chronic diseases. There are various secondary metabolites such as flavonoids, phenolic acids, lignans, quinones, coumarins, and alkaloids, which showed substantial antioxidant and other activities and have played an important role in the treatment of cancer (Boy et al., 2018). Natural compounds have shown immense potential against cancer as they are associated with minimal side effects and as a result, the use of plant-based natural compounds has garnered substantial focus. It is estimated that about 80% of the population in developing countries depends on natural or herbal medicine to treat different diseases (Purushotham et al., 2016). The presence of various secondary metabolites such as alkaloids, terpenoids, glycosides, steroids, flavonoids, and phenolic compounds in plants are responsible for their different pharmacological properties (Adiloğu, 2017). These bioactive compounds are reported to have various beneficial effects in decreasing the risk of diseases caused by reactive oxygen species (ROS) (Zilani et al., 2017) through different mechanisms of action such as scavenging free radicals, quenching ROS, and inhibiting oxidative enzymes. Reports have established that plant extracts have a range of biochemical properties such as antiallergic, anti-inflammatory, antioxidant, antimicrobial, antifungal, antiviral, and anticancer (Pisoschi and Pop, 2015).

Oxidative stress is recognized as an imbalance between ROS generation and their elimination by protective mechanisms, which can lead to chronic inflammation. Reports have demonstrated that oxidative stress plays a pathogenic role in chronic inflammatory diseases. Moreover, ROS have been highlighted as one of the main causes of several inflammatory diseases such as cardiovascular diseases (CVD), type II diabetes, and cancer (Hussain et al., 2016).

*Adenium obesum* (Forssk) Roem and Schult is commonly called “desert rose” and belongs to the family Apopcynaceae and has earlier been reported for its anti-influenza (Kiyohara et al., 2012), antimicrobial (Hossain et al., 2017), and cytotoxic efficacy (Almehdar et al., 2012). It is a rich reservoir of cardiac glycosides which contains around 50 phytochemicals belonging to the class of cardenolides, flavonoids, triterpenes, and pregnanes (Versiani et al., 2014). Different parts of this plant are traditionally used for treatment of various diseases including wounds, skin diseases, joint pain, and muscle pain in the Middle-East region (Hossain and Rahman, 2011). *A. obesum* is a rich source of cardiac glycosides, which showed a broad spectrum of biological activities. About fifty chemical constituents have been isolated from different parts of this plant. These compounds belong to different classes of cardenolides, pregnanes, triterpenes, flavonoids, and one carbohydrate.

In this study, we investigated the *in vitro* antioxidant, anticancer, and anti-inflammatory activities of the ethanolic extracts obtained from leaves of AOE.

## 2 Materials and Methods

### 2.1 Chemicals

α,α_-diphenyl-_-picrylhydrazyl (DPPH) and 2,2_-azino-bis (3-ethylbenzthiazoline-6-sulphonic acid) (ABTS) were purchased from Sigma–Aldrich (St. Louis, MO). Ascorbic acid (vitamin C), gallic acid, rutin, nitro blue tetrazolium (NBT), and butylated hydroxytoluene (BHT) were purchased from Hi-Media, India. The remaining chemicals and solvents used were of standard analytical grade and HPLC-grade. 2, 7-dichlorodihydrofluorescein diacetate (DCFH-DA) and Hoechst 33342 dye were obtained from from Sigma (St. Louis, MO, United States ). DMEM-high glucose medium, fetal bovine serums (FBS), and antibiotic–antimycotic solution were procured from Himedia India, Ltd., Mumbai, India, whereas a colorimetric kit specific for caspase-8, -9, and -3 was provided by BioVision, CA, United States . The DyNAmoColorFlash SYBR Green qPCR Kit (F415L) along with the Verso cDNA synthesis kit were procured from Thermo-Scientific, United States . The primers used in the present work were synthesized and purchased from IDT, United States .

### 2.2 Plant Collection

#### 2.2.1 Extraction

The leaves of *A. obesum* were initially washed with running water and shed-dried for about 6–7 days and then cut and crushed separately in a grinder. The powdered form is obtained and stored in dry tubes. Thereafter, the powdered samples were packed in a Soxhlet apparatus and extracted with 150 ml of 70% ethanol and 30% water as it showed the best extraction yield (Hossain and Shah, 2015). Thereafter, the resultant mixture was filtered using an evaporator (40°C). Subsequently, the extract was dried under Telstar Cryodos and stored at –20 °C for further analysis. The resultant semi-solid ethanolic extract of *A. obesum* (AOE) was stored at 4°C. The extraction yield (%) was calculated as follows (Motadi et al., 2020):

Extract yield % = Weight of the extract after evaporating solvent and freeze drying/Dry weight of the sample × 100.

#### 2.2.2 Gas-Chromatography Coupled With Mass Spectroscopy (GC-MS) Analysis

Characterization of the secondary metabolites present in the ethanolic leaf extract of *A. obesum* was carried out through GC-MS as described previously (Kushwaha et al., 2019). The extract was dissolved in 100% ethanol prior to analysis, and the experiment was performed on a Shimadzu QP 2010 Ultra-Mass Spectrometer with an RTXi-5MS column. The parameters during the analysis have been appended in Table 1.

**TABLE 1:** Parameters used during GC-MS analysis.

Parameters	Range
Ion source temperature	200 °C
Interface temperature	260 °C
Pressure mode	66.7 kPa
Total flow rate	10.4 ml/min
Column flow rate	1.24 ml/min
Column injector temperature	250 °C
Column oven temperature	280 °C

The sample was delivered within the instrument *via* an injector in the split mode in presence of helium. The identification of the bioactive constituents within the extract was accomplished by evaluating the retention time and fragmentation pattern through NIST11.0 spectral library and GC-MS Real-Time Analysis Software version 1.10 beta.

### 2.3 Cell Line Maintenance

Human lung cancer (A549) cells and J774A.1 murine macrophages were procured from the repository of the National Centre for Cell Sciences (NCCS), Pune, India. Both the cells were maintained in DMEM-high glucose completed with 10% FBS and 1% antibiotic–antimycotic solution in a controlled humidified atmosphere at 37°C with at least 5% CO_2_.

### 2.4 *In Vitro* Antioxidant Activity

#### 2.4.1 DPPH Radical Scavenging Assay

1,1-diphenyl 1-2-picryl-hydrazyl (DPPH) assay was used to determine the free radical scavenging activity (Munro et al., 2015). In order to evaluate the percent (%) antioxidant activity, 1 ml of plant extracts (50, 100, 200, 300, and 400 μg/ml) was mixed with 3 ml of the DPPH solution (100 mM). These reaction mixtures were shaken vigorously and incubated for 30 min in dark, and the decrease in absorbance due to proton-donating activity of AOE was recorded at 517 nm using a UV–Visible spectrophotometer. Methanolic DPPH of equal volume was used as the blank control. Ascorbic acid was used as a positive control. Radical scavenging activity was calculated as follows:

$$\mathrm{DPPH}\;\mathrm{radical}\;\mathrm{scavenging}\;\mathrm{rate}=\left[\left(A_{0}-A_{1}\right)\right]/A_{0}\;X\;100,$$

where A_0_ is the absorbance of pure DPPH or control and A_1_ is the absorbance of DPPH in the presence of various extracts or ascorbic acid.

#### 2.4.2 ABTS Radical Scavenging Activity

2,2′-azino-bis (3-ethylbenzothiazoline- 6-sulfonic acid) (ABTS) cation decolorization assay is similar to the DPPH assay, and it was also performed for the measurement of antioxidant potential of the plant extracts by using standard protocols described earlier with slight modifications (Liyanaarachchi et al., 2018). 1 ml aliquot of plant extract (50, 100, 200, 300, and 400 μg/ml) was prepared and mixed with 3 ml of ABTS working stock and subsequently incubated for 10 min in dark. Thereafter, the absorbance was taken at 734 nm. 50% methanolic ABTS was used as the control and ascorbic acid was used as a reference standard. The scavenging rate of ABTS was calculated by using the aforementioned formula in Section 2.4.1.

#### 2.4.3 Superoxide Radical Scavenging Activity

The superoxide radical scavenging activity was evaluated as described previously (Robak and Gryglewski, 1988). Superoxide radicals were produced in a PMS-NADH system *via* the oxidation of NADH and estimated through the reduction of NBT. In the present experiment, the superoxide radicals were produced in 3 ml of sodium phosphate buffer (100 mM, pH 7.4) containing 1 ml of NBT (150 mM) solution, 1 ml of NADH (468 mM) solution, and different doses of the AOE (50, 100, 200, 300, and 400 μg/ml) in water. This is followed by the addition of 1 ml PMS solution (60 mM) to the mixture, and the reaction mixtures were incubated for 5 min at room temperature. The absorbance was calculated against the corresponding blank solution. Ascorbic acid was used as the reference standard. The decline in the extent of NBT reduction, as evaluated by the absorbance of the reaction mixture, correlates with the superoxide radical scavenging activity of the AOE extract. The percentage of superoxide radical scavenging was measured by using the aforementioned equation.

#### 2.4.4 Hydroxyl Radical Scavenging Activity Assay

The scavenging activity for hydroxyl radicals was evaluated with the Fenton reaction as described previously by Yu et al. (Wenli et al., 2004). The reaction mixture contained 60 ml of 1.0 mM FeCl_2_, 90 ml of 1 mM 1,10-phenanthroline, 2.4 ml of 0.2 M phosphate buffer (pH 7.8), 150 ml of 0.17 M H_2_O_2_, and 1.5 ml of AOE extract at different concentrations. Addition of H_2_O_2_ initiated the reaction. Thereafter, reaction mixtures were incubated for 5 min, and the absorbance of the mixture was measured at 560 nm with a spectrophotometer. The scavenging of hydroxyl radicals was calculated by the aforementioned equation.

#### 2.4.5 Lipid Peroxidation Assay

Non-enzymatic Fe^3+^/ascorbic acid–mediated lipid peroxidation in bovine brain extract was performed as described previously (Houghton et al., 1995) with subtle modification. The reaction was constituted by 50 µl bovine brain phospholipids (5 mg/ml), FeCl_3_, and ascorbic acid (1 mM each dissolved in 20 mM PBS) in the presence and/or absence of AOE (50,400 μg/ml) or the reference compound. The final volume of the reaction was 330 µl, and the reaction was briefly incubated for 1 h at 37°C. The generation of hydroxyl radicals provided the impetus for lipid peroxidation, which further augmented the synthesis of malondialdehyde (MDA). MDA was subsequently quantified with the TBA reaction through ELISA reader at 532 nm. Ascorbic acid during the study served as a positive control. The results were expressed as percentage inhibition activity calculated as.

$\text{Inhibition}\;\text{activity}\;\text{percentage}=\left({\text{A}}_{\text{Control}}{\;-\text{ A}}_{\text{Test}}\right){\text{/ A}}_{\text{Control}}\;\text{X}\;100\text{,}$ where A_Control_ was the absorbance of the control and A_Test_ was the absorbance of the sample.

### 2.5 *In Vitro* Anticancer Activity

#### 2.5.1 Cell Viability Assay

In order to study the cytotoxic potential of AOE, an MTT assay was performed as per the earlier described protocol with slight modifications (Ahmad et al., 2021a). Briefly, 5 × 10^3^ A^54^9 cells/well were seeded in a 96-well plate and allowed to adhere in a humidified atmosphere. A549 cells were treated with different concentrations of AOE (100, 200, and 400 μg/ml) and further incubated for 24 h under standard conditions. MTT dye (10 μl; 5 mg/ml) was added to all the wells and further incubated for 4 h (37 °C). Finally, the formazan or purple-colored crystals were solubilized by supplementing 100 μl DMSO. Thereafter, each well was assessed for absorbance of formazan crystals at 490 nm using a microplate reader (Bio-Rad, California, United States ), and the cell viability was calculated as percent (%) cell viability in comparison with the untreated control.

#### 2.5.2 Phase Contrast Microscopy

Phase-contrast microscopy was performed to study the morphological alterations within the AOE-treated A549 lung cancer cells. Cells were incubated overnight in a 96-well plate approximately and cultured with different concentrations of AOE for 24 h. Morphological alterations within the AOE-treated A549 cells along with the control group were visualized and captured using the relief phase channel of the FLoid imaging station at ×20 magnification (Thermo-Scientific, United States).

#### 2.5.3 Evaluation of Nuclear Condensation

Hoechst 33342 dye assay was used to study the nuclear condensation in AOE-treated A549 lung cancer cells (Ahmad and Ansari, 2021). A549 cells (5 × 10^3^ cells/well) were seeded and allowed to adhere and thereafter exposed to AOE treatment (24 h) at different concentrations as mentioned earlier and incubated under standard conditions. After that, the media were decanted, and cells were further stained with Hoechst 33342 dye (2 μg/ml; 10 min) under standard conditions. Last, fluorescent nuclei of treated cells were visualized, and photomicrographs were captured using a blue fluorescence channel (Excitation: 390/40 nm–Emission: 446/33 nm) of FLoid imaging station (Thermo-148 Scientific, United States).

#### 2.5.4 DCHF-DA Staining Assay

ROS generation in AOE-treated A549 lung cancer cells was qualitatively analyzed by using DCHF-DA staining as described earlier (Ahmad et al., 2021b). The cells were allowed to adhere overnight in a 96-well plate under standard conditions. After incubation, cells were exposed to different concentrations of AOE for 24 h. DCFH-DA (10 μM) was added per well after decanting media and incubated in dark for 30 min at RT. Finally, the cells were washed using PBS to remove excess stain if any prior to imaging using a green fluorescence channel (excitation: 482/18 nm–emission: 532/59 nm) of FLoid imaging station (Thermo-Scientific, United States ).

#### 2.5.5 Evaluation of Caspase Activity

A colorimetric caspase assay kit specific for caspase-9 and -3 was used as per the manufacturer’s instructions. The results were interpreted as a percentage increase in the activity of specific caspases in comparison with the control cells.

#### 2.5.6 Real-Time PCR

Approximately 1 million A549 cells were exposed to AOE (100, 200, and 400 μM) treatment for 24 h followed by total RNA extraction using a commercial grade kit according to the manufacturer’s protocol. 2 μg of extracted RNA was further used for cDNA synthesis by using the Verso cDNA synthesis kit as per the protocol. The primer sequences (0.5 µM each of forward and reverse primers) used in this study are listed below as described previously (Ahmad et al., 2021b). Furthermore, PCR assay was performed using the DyNAmoColorFlash SYBR Green qPCR Kit in accordance with the manufacturer’s instructions.

**Table:** 

GAPDH	CGACCACTTTGTCAAGCTCA	CCCCTCTTCAAGGGGTCTAC
Bax	GCTGGACATTGGACTTCCTC	CTCAGCCCATCTTCTTCCAG
Bad	CCTCAGGCCTATGCAAAAAG	AAACCCAAAACTTCCGATGG
Bcl2	ATTGGGAAGTTTCAAATCAGC	TGCATTCTTGGACGAGGG

### 2.6 *In Vitro* Anti-inflammatory Activity

#### 2.6.1 ELISA-Based Estimation of Inflammatory Mediators

Nearly 10^6^ J774A.1 cells/well were transferred to a 6-well plate and stimulated with or without 100 ng/ml LPS for 24 h. Post simulation, the cells were exposed to varying concentrations of *A. obesum* ethanolic leaf extract (100, 200, and 400 μg/ml) for an additional 24 h under standard culture conditions. Subsequently, the levels of inflammatory mediators, namely, IL-1β, TNF-α, IL-10, and PEG2 were quantified through commercially available ELISA kits (BD Bioscience, CA, United States ) in accordance with the manufacturer’s protocol. Cells with and without LPS stimulation served to be positive and negative control, respectively.

### 2.7 Statistical Analysis

All reported data are expressed as the mean ± SEM of three individual experiments performed thrice through GraphPad Prism (Ver. 5). Statistical analysis among different treatment groups was determined using one-way ANOVA followed by Dunnett’s post-test. Significance: ∗*p* < 0.05, ∗∗*p* < 0.01, and ∗∗∗*p* < 0.001.

## 3 Results

### 3.1 Gas Chromatography-Mass Spectroscopy (GC-MS) Analysis

**FIGURE 1:**
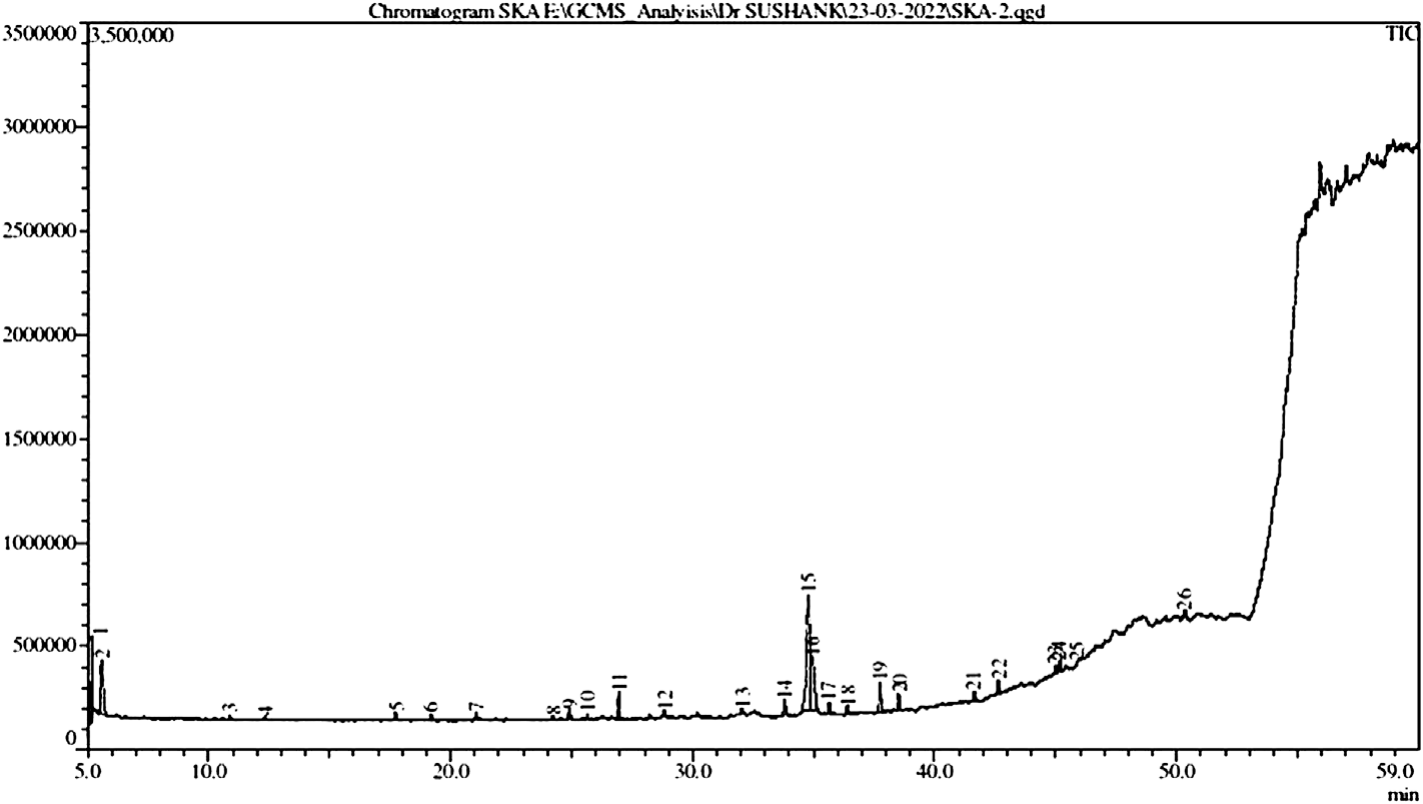
GC-MS chromatogram of the ethanolic extract of *Adenium obesum* leaves.

The GC–MS chromatogram of ethanolic extracts of *A. obesum* (Figure 1) recorded a total of 26 peaks corresponding to the bioactive compounds that were recognized by evaluating the retention time and fragmentation pattern through the NIST11.0 spectral library and GC-MS Real-Time Analysis. The phytoconstituents recognized have been included in Table 2. The chromatogram of the extract explicitly showed the presence of several phytoconstituents which were present in various extracts that have been associated with anticancer activity against different carcinomas. Tetraethylene glycol, 1-Pentadecane, heptadecane, 2-benzenedicarboxylic acid, dodecane, pentaborane, trichloro methane, 1-Dodecene, tetradecane, 2-ethyl-1-hexanol, heptadecane, dimethyl sulfoxide, 1-Pentadecene, phosphoric acid, triethyl ester, nitro-benzene, 2-(2-butoxyethoxy)-acetate ethanol, 1,2,4,5-tetrachlorobenzene, etc were found in the ethanolic extract of AOE.

**TABLE 2:** List of different phytochemicals present within the ethanolic leaf extracts of *Adenium obesum* as recognized through GC-MS analysis.

Peak No.	Retention time	Area (%)	Height (%)	Name of the constituent
1	5.175	3.17	16.26	Pentaborane
2	5.581	11.80	10.32	Trichloro methane
3	10.870	0.85	0.98	Dodecane
4	12.336	0.44	0.67	1-Dodecane
5	17.733	0.84	1.24	Tetradecane
6	19.210	0.77	1.29	1-Pentadecane
7	21.067	0.87	1.36	2-ethyl-1-hexanol
8	24.238	0.60	0.82	Heptadecane
9	24.907	1.58	1.91	Dimethyl sulfoxide
10	25.651	0.63	1.05	1-Pentadecane
11	26.948	3.63	5.21	Phosphoric acid, triethyl ester
12	28.835	0.79	1.10	Nitro-benzene
13	32.037	0.73	1.01	2-(2-butoxyethoxy)-acetate ethanol
14	33.807	2.40	2.89	1.2,4,5-Tetrachlorobenzene
15	34.979	39.16	22.34	1,2-Benzenedicarboxylic Acid
16	34.968	13.44	10.08	1,2-Benzenedicarboxylic Acid
17	35.649	1.47	2.10	Acrylate 1-Tetradecanol
18	36.391	1.39	1.59	Phenol
19	37.745	4.93	5.39	α-benzeneacetic acid
20	38.526	2.14	2.97	2-Butenedioic acid (Z)-, dibutyl ester
21	41.646	1.49	1.63	Methyl mandelate
22	42.636	2.32	2.61	1-Propanone
23	44.995	1.09	1.48	l-mandelate ethyl
24	45.151	1.81	1.97	1,2-Benzenedicarboxylic Acid
25	45.892	0.05	0.18	2-methyl-1,3-dioxolan-2-yl
26	50.335	1.59	1.32	Tetraethylene glycol

### 3.2 DPPH Radical Scavenging Potential of AOE

The DPPH is a stable and organic free radical with an absorption band at 512–528 nm primarily used for investigating the free radical scavenging activity of various compounds. DPPH assay is based on the reducing capability of alcoholic DPPH solution in the presence of hydrogen-donating stimulant (Sánchez-Moreno, 2002; Sharma and Gupta, 2007). The AOE was capable of neutralizing DPPH-free radicals via hydrogen-donating activity by 18.48%, 34.29%, 59.20%, 73.24%, and 86.37% at the indicated doses of 50, 100, 200, 300, and 400 μg/ml. As shown in Table 3, DPPH radical scavenging was increased in a dose-dependent manner as compared to ascorbic acid, used as the reference or positive antioxidant control in this study.

**TABLE 3:** DPPH, ABTS, superoxide, hydroxyl radical scavenging, and lipid peroxidation inhibition potential of AOE and ascorbic acid.

AOE (µg/ml)	DPPH Scavenging (%)	Superoxide Scavenging (%)	ABTS Radical Scavenging (%)	Hydroxyl Radical Scavenging (%)	Lipid Peroxidation Inhibition (%)
50	18.48 ± 0.94^a^	10.36 ± 0.95^a^	13.56 ± 1.26^a^	16.59 ± 1.39^a^	22.38 ± 1.58^a^
100	34.29 ± 0.99^a^	19.72 ± 2.15^a^	46.72 ± 1.39^a^	38.65 ± 1.85^a^	39.76 ± 1.25^a^
200	59.20 ± 2.61^a^	39.86 ± 2.31^a^	55.39 ± 2.72^a^	58.72 ± 2.11^a^	48.52 ± 2.21^a^
300	73.24 ± 2.93^b^	54.26 ± 2.56^b^	69.56 ± 2.83^b^	72.36 ± 2.42^b^	63.89 ± 2.52^a^
400	86.37 ± 1.93^b^	68.92 ± 1.96^b^	79.21 ± 1.89^b^	84.52 ± 2.62^b^	76.52 ± 2.86^a^
Ascorbic acid	96.12 ± 1.61^b^	84.75 ± 2.17^b^	95.72 ± 2.19^b^	98.56 ± 2.46^b^	82.84 ± 2.51^a^

Data reported here represent mean ± SEM (*n* = 3).

a *p* < 0.05.

b *p* < 0.01 comparatively with the control.

DPPH: 2,2-diphenyl-1-picrylhydrazyl; ABTS: 2.2′-Azino-bis(3-ethylbenzothiazoline-6-sulfonic acid) diammonium salt; AOE: adenium obesum extract.

### 3.3 ABTS Radical Scavenging Potential of AOE

The ABTS radical scavenging assay gives a specific absorbance at a wavelength of 734 nm from the visible region and requires a short reaction time, which could be used as an index that indicates the antioxidant activity of the reaction samples (Ilyasov et al., 2020). In Table 3, AOE was found to be very effective in scavenging ABTS radical, and the increase was dose-dependent by 13.56%, 26.72%, 55.39%, 69.56%, and 79.21% at the indicated concentrations of 50, 100, 200, 300 and 400 μg/ml. As shown in Table 1, ABTS radical scavenging was increased in a dose-dependent manner as compared to ascorbic acid, used as the reference or positive antioxidant control in this study. Thus, it is suggested that AOE holds a good capability to scavenge the ABTS radical.

### 3.4 Superoxide Anion Scavenging Potential of AOE

Reports have very well established that superoxide anions damage biomolecules either directly or indirectly by producing H_2_O_2_, peroxy nitrite, OHˉ, or singlet oxygen during aging and pathological events including ischemic reperfusion injury. It is also observed that superoxide has directly initiated lipid peroxidation (Yen and Duh, 1994; Ahmed et al., 2013). The superoxide anion radical scavenging activity of AOE assayed by the PMS-NADH system is demonstrated in Table 3. The superoxide scavenging activity of AOE was markedly increased by 10.36%, 19.72%, 39.86%, 54.56%, and 68.92% with increase in doses. Thus, AOE exhibited stronger inhibitory effects on superoxide anion formation noted herein, possibly rendering them a propitious antioxidant. These data substantiated that AOE has potent superoxide radical scavenging effects.

### 3.5 Hydroxyl Radical Scavenging Potential of AOE

Hydroxyl radical is a strongly reactive oxygen-centered radical formed as a by-product of the reaction of several hydroperoxides with transition metal ions. It attacks different biological molecules such as proteins, DNA, and polyunsaturated fatty acids in membranes and is also known to be capable of abstracting hydrogen atoms from membrane lipids (Yen and Duh, 1994) and carries out peroxidation of lipids. AOE showed dose-dependent scavenging activity against hydroxyl radicals generated in the Fenton reaction system by 16.59%, 38.65%, 58.72%, 72.36%, and 84.52%.

### 3.6 Lipid Peroxidation Inhibitory Activity

Lipid peroxidation was colorimetrically assessed by quantifying TBA at 532 nm. As shown in Table 3, AOE showed its competence in inhibiting the lipid peroxidation significantly by 76.50% (*p < 0.05*), whereas ascorbic acid also considerably inhibited the lipid peroxidation by 82.84%. The AOE-mediated inhibitory effect on lipid peroxidation was found to be dependent on the concentration of AOE.

### 3.7 AOE Exerts Cytotoxic Effects in A549 Lung Cancer Cells

**FIGURE 2:**
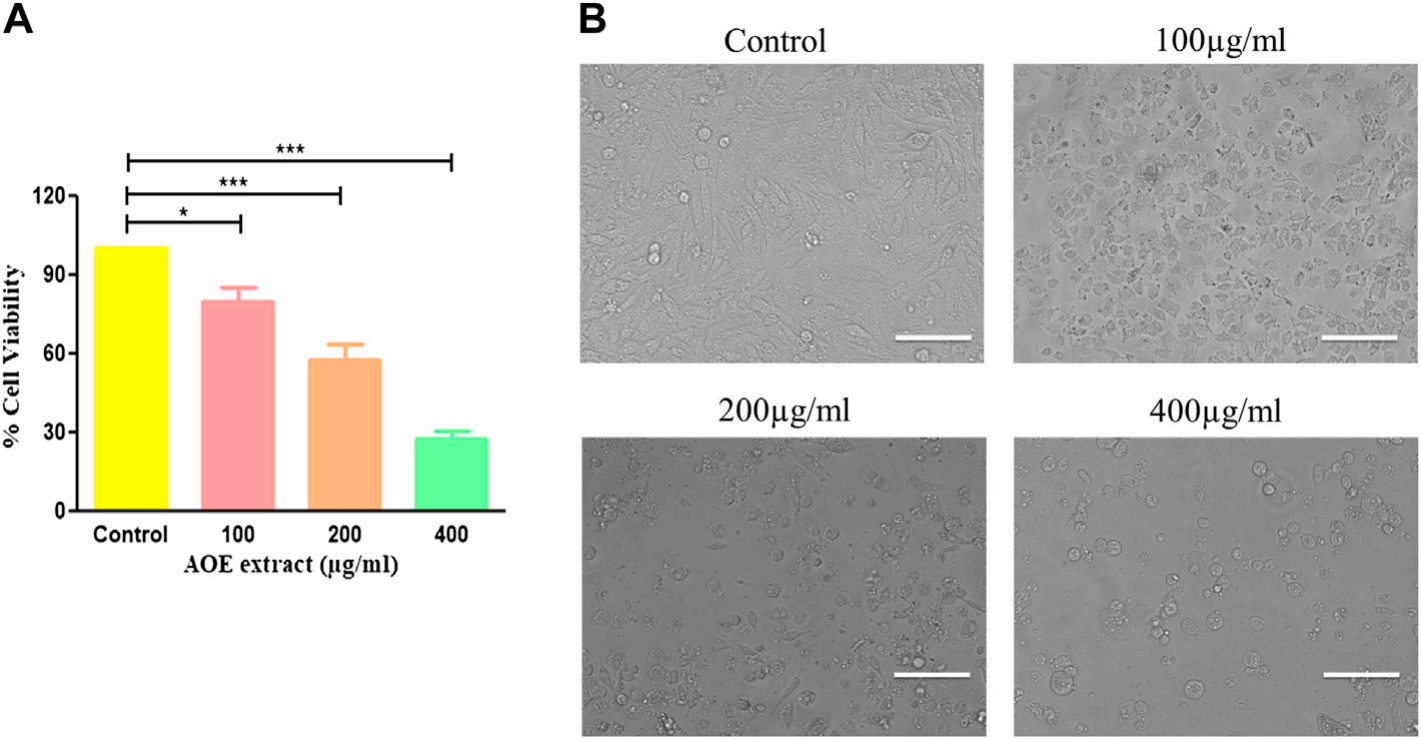
Effect of AOE on the growth of A549 lung cancer cells. **(A)** Percent (%) cell viability of A549 cells after AOE treatment at concentrations of 100–400 μg/ml after 24 h. **(B)** Phase-contrast images of AOE-treated A549 cells for 24 h. Data communicated constitutes the mean ± SEM of individual experiments performed thrice in triplicate. The level of significance among different AOE-treated groups was determined using one-way ANOVA and Dunnett’s post-hoc in comparison with the untreated control where *represents *p*<0.05, ***p<0.001; Scale bar = 100 μm, and magnification = 20X

In order to study the anticancer or cytotoxic effects of AOE on A549 lung cancer cells, an MTT assay was performed. A549 cells were treated with different concentrations of AOE (100, 200, and 400 μg/ml) for 24 h. The IC_50_ of AOE against A549 lung cancer cells was found to be IC_50_ ± 256.75 μg/ml. AOE treatment significantly decreased the cell viability of A549 cells as shown in Figure 2A, which was found to be 79.82% ± 4.39, 57.82 ± 4.83%, and 27.48 ± 2.52% as compared to untreated cells or control cells. Thus, AOE exhibited substantial cytotoxic activity against lung cancer cells.

### 3.8 AOE Induces Morphological Changes in A549 Lung Cancer Cells

Morphological analysis of AOE-treated lung cancer cells was undertaken using a phase contrast microscope. A dose-dependent morphological alteration was observed in AOE-treated A549 cells. Lung cancer cells undergo several morphological alterations including round morphology with slight shrinkage and nuclear condensation in the presence of different concentrations of AOE (100, 200, and 400 μg/ml). These morphological changes within lung cancer cells were more obvious with the increase in the dose of AOE, whereas control cells exhibited flattened morphology (Figure 2B).

### 3.9 AOE Mediates Nuclear Condensation in A549 Lung Cancer Cells

**FIGURE 3:**
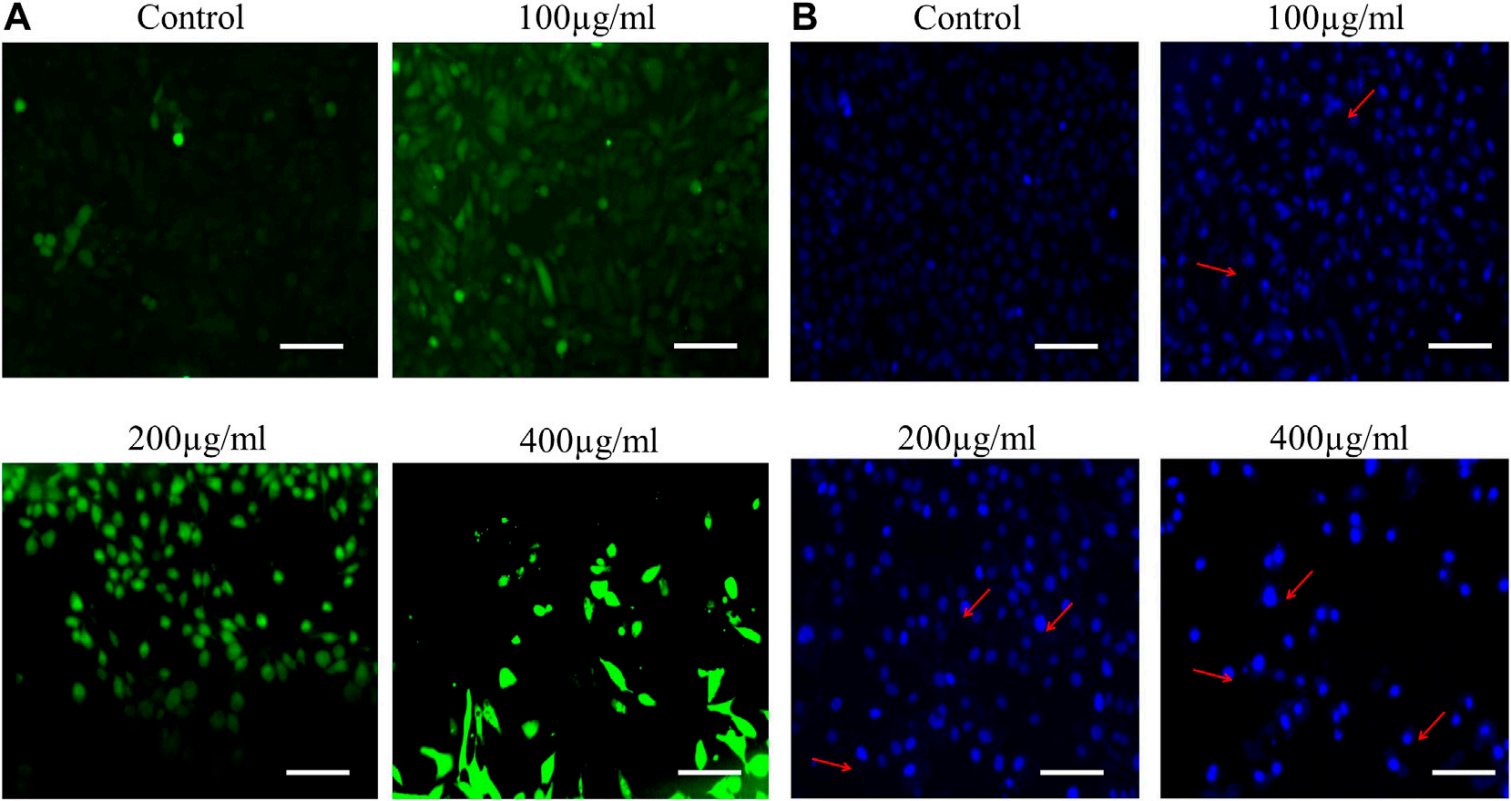
**(A)** AOE-mediated induction of apoptosis within treated human lung cancer A549 cells. Increased levels of nuclear condensation and apoptosis are depicted within photomicrographs of vehicle control and AOE-treated (100–400 µg/ml) A549 cells stained with Hoechst 33342 by red arrows. **(B)** Enhanced intracellular ROS generation in DCHF-DA-stained A549 cells treated with AOE for 24 h. Scale bar = 100 μm and magnification = 20X

Nuclear condensation and fragmentation are the two peculiar hallmarks of apoptosis. Hoechst 33342 staining was performed to qualitatively analyze whether the AOE-induced cytotoxicity in lung cancer cells was due to apoptosis induction. As observed from the fluorescent micrographs, treatment of AOE induces nuclear condensation and fragmentation in A549 cells in a dose-dependent manner as indicated by the white arrows, while the control cells exhibited unaltered morphology (Figure 3A).

### 3.10 AOE Augments ROS Generation in A549 Lung Cancer Cells

In order to study the effect of AOE on ROS, we perform DCFH-DA staining. A549 cells were stained with DCFH-DA to detect the changes in the levels of intracellular ROS after 24 h treatment of various concentrations of AOE. The fluorescent micrographs exhibited that treatment with different doses of AOE (100, 200, and 400 μg/ml) resulted in stronger DCF-fluorescence intensity in lung cancer cells, which indicated an enhanced intracellular ROS generation caused by AOE (Figure 3B).

### 3.11 AOE Increases the Activity of Caspases in A549 Lung Cancer Cells

**FIGURE 4:**
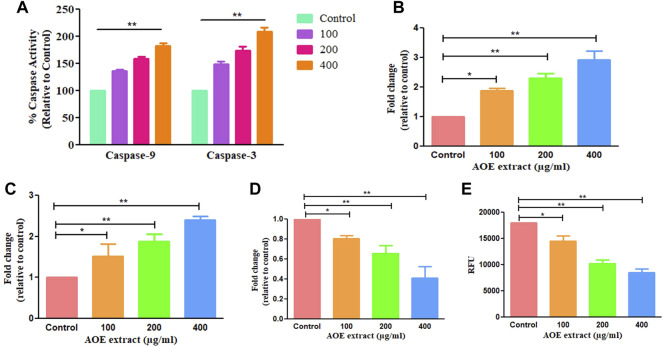
AOE treatment-induced consequences on activation of caspase-9 and -3 within A549 cells **(A)** percent (%) activation of caspase-9 and -3 activities post AOE exposure at 100–400 µg/ml concentrations, **(B-E)** mRNA expression levels of pro- and anti-apoptotic genes within AOE-treated A549 cells. **(E)** Decrease in NIR fluorescence in AOE-treated cells after staining with Mito-NIR dye, suggesting depolarization of ΔΨ_m_.

Bcl-2 family members and caspases are primarily responsible for regulating apoptosis or programmed cell death in multicellular organisms. It is subdivided into two types: extrinsic (death receptor) and intrinsic (mitochondrial) pathways. As a result, we performed caspase assay on AOE-treated A549 cells, and it was observed that AOE (100–400 μM) treatment significantly increased the caspase-9 and -3 activity by 36.31 ± 2.31%, 58.39 ± 4.02%, and 82.25 ± 4.29% and 49.03 ± 3.96%, 74.46 ± 5.47%, and 109.20 ± 5.56% respectively (Figure 4A).

### 3.12 AOE Modulates the Expression of Bcl-2 Family Members in A549 Lung Cancer Cells

We studied the mRNA levels of Bax, Bad (proapoptotic protein), and Bcl-2 (antiapoptotic protein) which are chiefly involved in mediating the mitochondria-dependent apoptotic pathway. The mRNA levels of Bax, Bad, and Bcl-2 in AOE-treated A549 cells were measured through qRT-PCR analysis. The mRNA levels of Bax and Bad enhanced to 1.88 ± 0.07, 2.30 ± 0.13, and 2.92 ± 0.24 folds and 1.52 ± 0.24, 1.88 ± 0.13, and 2.40 ± 0.06 folds respectively, comparatively with the untreated cells (Figures 4B,C). However, Bcl-2 (antiapoptotic protein) mRNA declined to 0.87 ± 0.02, 0.65 ± 0.06, and 0.41 ± 0.09 folds comparatively with the control (Figure 4D). Thus, AOE treatment increased the expression level of pro-apoptotic proteins and decreased the expression level of anti-apoptotic proteins in A549 lung cancer cells.

### 3.13 AOE Alters Mitochondrial Membrane Potential in A549 Lung Cancer Cells

Mitochondria play an important role in inducing apoptosis by mitochondrial or intrinsic apoptotic pathways. As shown in Figure 4E, decrease in the fluorescence of NIR was observed in AOE-treated cells after staining with Mito-NIR dye, indicating depolarization of mitochondria as compared with the control where the mitochondria appeared to be intact. Thus, AOE treatment significantly altered MMP directly depending upon the AOE dose in lung cancer cells.

### 3.14 AOE Ameliorated LPS-Induced Inflammatory Mediators

**FIGURE 5:**
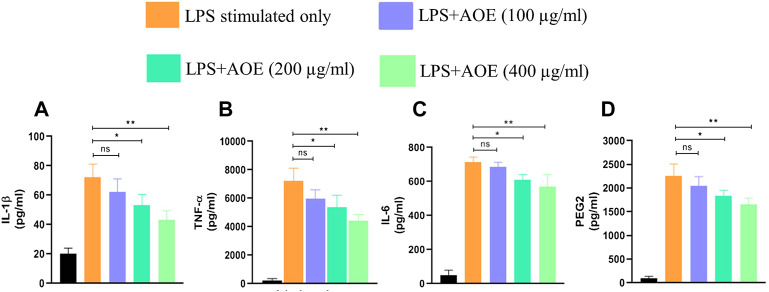
Potential of AOE in deflating the LPS-mediated inflammatory response as characterized by the levels of **(A)** IL-1β, **(B)** TNF-α, **(C)** IL-6, and **(D)** PGE2 within J774A.1. . Data communicated constitute the mean ± SEM of individual experiments performed thrice in triplicates. The level of significance among different AOE-treated groups was determined using one-way ANOVA and Dunnett’s post-hoc in comparison with untreated control where *represents *p*<0.05 and **p<0.01

Proinflammatory cytokines, namely, TNF-α, IL-6, and IL-1β in the supernatant of respective groups were substantially elevated post stimulation with LPS, which is an established instigator of the inflammatory response (Figure 5). The ethanolic leaf extract of *A. obesum* substantially reduced the level of TNF-α, IL-6, and IL-1β in a dose-dependent manner (*p* < 0.05, *p* < 0.01). Similar effects were observed on PGE2 levels within J774A.1 cells treated with ethanolic leaf extract of *A. obesum* again in a dose-dependent manner (Figure 5).

## 4 Discussion

Cancer remains a leading cause of mortality worldwide in spite of considerable advancement and progression in basic research and clinical studies. Early diagnosis and chemoprevention are key essentials for declining the incidence of cancers. In addition, the side effects of conventional therapeutics contribute to diminishing patient life quality and imply the need to develop a safe and effective therapeutic modality. Even though studies have been conducted to combat cancer in terms of natural therapy, a satisfactory and complete therapeutic agent has not been found.

ROS are accountable for the damage of various cellular biomolecules including proteins, nucleic acids, enzymes, lipids, and carbohydrates and may severely affect the functions of the immune system (Nilsson et al., 2004). Antioxidants intrude on the generation of ROS and also play a crucial role to inactivate them. However, all the cells present in the human body protect themselves against oxidative damage by some antioxidant mechanism. Still, these mechanisms are not sufficient enough to prevent the ROS damage totally. Various kinds of plant extracts and their isolated compounds have been reported as natural antioxidants (Ljubuncic et al., 2006; Liao et al., 2008).

In the present study, the investigation of ethanolic extracts from leaves of *A. obesum* revealed the presence of various phytoconstituents such as carbohydrates, flavonoids, glycoside, cardiac, prenylated flavonoids, terpenoids, pregnanes, etc. In the present study, we have examined the antioxidant, anticancer, and anti-inflammatory properties of the ethanolic leaf extracts of *A. obesum*. These bioactive phytoconstituents could be accountable for the therapeutic ability of ethanolic extracts of *A. obesum*. The GC–MS analysis of *A. obseum* leaf extracts revealed the presence of 26 phytochemical compounds, which could be associated with the medicinal properties of this plant species. It was recently found that tetraethylene glycol exerts anticancer effects against Jurkat, K562, HEK293, HeLa, and U937 cell lines (D’yakonov et al., 2021). 1-pentadecane was also recently reported to be present in ethyl acetate extract of *S. chamaecyparissus* which showed antidiabetic and anticancer efficacy against human breast cancer MCF-7 cells (Ali et al., 2021). 1-pentadecane was also reported to be present in *Streptomyces malaysiense* sp. nov. actinobacteria and exhibited substantial cytotoxic effects against human colon cancer HCT-116 cells (Ser et al., 2016). Heptadecane present within the ethanolic extract of *A. obesum* is a volatile component of Spirulina platensis and is reported to have antiproliferative efficacy against human liver cancer HepG2 cells (Wu et al., 2005). *Allium willeanum* Holmboe extract was reported previously for the presence of 1,2-benzenedicarboxylic acid. This extract showed substantial anticancer efficacy against human breast cancer MCF-7 and MDA-MB-231 (Isbilen and Volkan, 2021). 1,2-benzenedicarboxylic acid was also reported to be present within the methanolic bark extract of *Berberis hispanica* and also showed anticancer potential against human breast cancer (MCF-7 and MDA-MB-231 cells) and human prostate cancer LnCap and 22 RV1 cells (El Fakir et al., 2021). Similarly, dodecane was also reported from the GC-MS analysis of endophytic fungi *Talaromyces purpureogenus* extracts and showed its anticancer efficacy against HPV18 + human cervical cancer HeLa cells (Kumari et al., 2018).

Initially, we performed a DPPH assay to investigate the antioxidant potential of AOE, and we observed dose-dependent scavenging of the DPPH radical. In addition, the reaction between ABTS and potassium persulfate leads to production of a blue-colored chromophore (ABTS*•* ^+^). This is followed by the addition of AOE to this pre-formed radical cation which was converted to ABTS in a concentration-dependent manner. The result is analogous to the earlier mentioned DPPH assay, suggesting that AOE acts as a potent antioxidant.

Hydroxyl radical is considered one of the ROS primarily formed in living systems, causing breaks in DNA strands, leading to carcinogenesis, cytotoxicity, and mutagenesis (Packer et al., 1997). We observed that the addition of AOE to the reaction mixture eradicates hydroxyl radicals and prevents subsequent damage.

Superoxide anion is also regarded as harmful ROS. It has a deleterious effect on various cellular components in a living system (Halliwell et al., 1987). It initiates lipid oxidation by producing singlet oxygen. The concentration-dependent increase in the scavenging activity of the AOE and the standard ascorbic acid for superoxide radical suggest that the AOE is a more potent scavenger than ascorbic acid (reference standard).

Lipid peroxidation is the main cause of food deterioration, affecting the color, flavor, texture, and nutritional value of food and food products. During the process of lipid peroxidation, free radicals steal electrons from the lipids in cell membranes, which may result in loss of membrane fluidity, increase in membrane permeability, and decrease in physiological performance, thus endangering cell viability (Bajpai et al., 2014). In the present study, the potential efficacy of AOE in inhibiting lipid peroxidation induced *via* the Fe3+/ascorbate system was measured in bovine brain extract. AOE at the concentration of 400 μg/ml showed 76.52% inhibitory effects on lipid peroxides.

Earlier published data on the effect of AOE extracts on cancerous cells are limited, and the mechanisms are not yet fully deciphered. In our present study, apoptosis induction was the central motif and from the results, we observed changes in the morphology of the cells upon treatment with AOE extracts. The lung cancer cells (A549) exhibited cell shrinkage, spikes, and other attributes resembling DNA fragmentation; these characteristics were similar to those reported in the past as evidence that cells are undergoing apoptosis 11–15. Our results suggested that AOE induced cell death in A549 cancer cells *via* apoptosis by instigating nuclear fragmentation and condensation as visualized through Hoechst 33342 staining. Moreover, we also found that AOE significantly increased the expression of apoptotic proteins (Bax and Bad) and decreased the expression of anti-apoptotic proteins (Bcl-2) in lung cancer.

Caspases are the crucial regulators of apoptosis and are family members of the cysteine proteases (Wong, 2011). Our results demonstrated that AOE increases the activities of caspases along with the modulation in the expression of proteins associated with apoptosis. Mitochondria are the main cellular sites involved in ROS generation, leading to mitochondrial dysfunction and release of cytochrome-c (Miller and Zachary, 2017). We observed a significant alteration in ΔΨ_m_ after treatment with AOE. We found that the AOE enhances the level of ROS, significantly mediating apoptosis.

Oxidative stress (OS) in the cells arises due to an imbalance between the formation and elimination of oxidant species (Pizzino et al., 2017). Reactive oxygen species (ROS) are the by-products of OS and they are unstable, small active molecules containing superoxide anion radicals, hydrogen peroxide (H_2_O_2_), singlet oxygen, and hydroxyl radicals. Moreover, increasing evidence suggests that escalated production of intracellular ROS may directly or indirectly induce damage to nucleic acids, proteins, and lipids, which eventually leads to induction of apoptosis (Das and Roychoudhury, 2014).

The acute inflammatory response is a prerequisite for homeostatic functioning of the innate immune response and bridging it with the adaptive arm of immunity. Indeed, chronic activation of inflammatory response results in onset of diseases such as arthritis and neurodegenerative disorders (Scott et al., 2010; Tiwari et al., 2021). Chronic inflammation within the tissue and/or organ of an individual results from the accumulation of inflammatory mediators including proinflammatory cytokines such as interferon (IFN)-γ, interleukin (IL)-1β, IL-6, and TNF-α [24876674]. In the herewith presented report, we further explored the anti-inflammatory potential of AOE in murine alveolar macrophages (J774A.1) as an *in vitro* screening system. The cells were stimulated with LPS which has been established for its potential to instigate the TLR-4-NF-κB–mediated inflammatory axis within these cells (Litak et al., 2020). AOE showed its potential in reducing the levels of inflammatory mediators including proinflammatory cytokines and TNF-α.

## 5 Conclusion

In conclusion, our present investigation has shown that AOE exhibited antioxidant, anticancer, and anti-inflammatory properties. AOE exhibited powerful antioxidant activity evaluated *in vitro*, suggesting that AOE could be a reservoir of natural antioxidants. Our findings open up the possibility in the future to identify the potential therapeutic agents from AOE for the development of herbal-based medicine.

## Data Availability

The original contributions presented in the study are included in the article/Supplementary Material; further inquiries can be directed to the corresponding authors.
